# Risk prediction model of polypharmacy for community-dwelling elderly patients: An assessment tool for early detection

**DOI:** 10.3389/fphar.2022.977492

**Published:** 2022-11-10

**Authors:** Qi Tang, Jing Lu, Wenhui Wu, Zhenwei Liu, Sitang Zhao, Chengyue Li, Gang Chen, Jun Lu

**Affiliations:** ^1^ School of Public Health, Fudan University, Shanghai, China; ^2^ China Research Center on Disability, Fudan University, Shanghai, China; ^3^ Key Laboratory of Health Technology Assessment, National Health Commission, Fudan University, Shanghai, China

**Keywords:** elderly, polypharmacy, risk prediction model, nomogram, family physicians

## Abstract

**Background:** Polypharmacy has become a major and growing public health issue, with significant implications for health outcomes and expenditure on healthcare resources. In this study, a risk prediction model of polypharmacy represented by a nomogram for community-dwelling elderly patients based on the Chinese population was constructed.

**Methods:** A cross-sectional study was conducted in Shanghai, China. The variables data affecting polypharmacy were fetched from the information system database of health government departments in Shanghai. The Least Absolute Shrinkage Selection Operator (LASSO) regression analysis was used to select the predictor variables, and multivariate logistic regression was used to establish the prediction model. A visual tool of the nomogram was established for predicting the risk of polypharmacy in the elderly population. In addition, the receiver operating characteristic (ROC) curve, calibration curve, and decision curve analysis (DCA) were used to estimate the performance of the model.

**Results:** A total of 80,012 elderly patients were included in this study. Eight variables, containing age, residential area, preferred medical institutions, number of visits to tertiary hospitals, number of visits to secondary hospitals, number of visits to community health centers, number of diagnoses, and main types of disease, were included in the risk prediction model of nomogram. The area under the curve (AUC) of the nomogram was 0.782 in both sets, demonstrating that the model has a good discriminant ability. The calibration chart shows that the prediction model fits well with the validation set. DCA results displayed that the threshold probabilities of the two sets in the prediction model reached up to 90%, implying that the model had a preferable application value.

**Conclusion:** This study explored the risk factors for polypharmacy among the elderly in Shanghai, China, and applied the nomogram to establish a predictive model *via* eight variables, which provided an effective tool for early screening and timely prevention of polypharmacy. Family physicians or pharmacists could scientifically use the tool to closely observe community-dwelling elderly patients, decreasing the adverse health effects caused by medication for the elderly.

## Introduction

The concurrent use of multiple medications, also referred to as polypharmacy, was most commonly applied to situations where patients took 5 or more medications, which is normal among elderly patients with multimorbidity (Masnoon et al., 2017). Polypharmacy increases the risk of adverse effects, like mortality, falls and adverse drug reactions, with significant implications for health outcomes and expenditure on healthcare resources (Gurwitz et al., 2003; Viktil et al., 2007; Cahir et al., 2010; Kojima et al., 2012). In the U.S., more than 50% of elderly patients with Medicare take 5 or more medications (Tinetti et al., 2004). In China, the average number of medication for the elderly with multimorbidity was 9.1, with the highest up to 36 (Endocrinology and Metabolism Branch of Chinese Association of Geriatric Research and Committee of Clinical Toxicology of Chinese Society of Toxicology, 2018). Other studies showed that Chinese elderly patients with polypharmacy had an average of 10.3 ± 5.1 kinds of drugs, and the proportion of polypharmacy in the elderly aged 80 and above reached 82.4% (Lai et al., 2016; Liu et al., 2017; Tao et al., 2021). The World Health Organization (WHO) estimated that mismanaged polypharmacy accounts for 4% of total avoidable costs globally due to improper medicine use (World Health Organization, 2019). Polypharmacy has become a major and growing public health issue in all healthcare settings around the world (Payne and Avery, 2011; Duerden et al., 2013).

Given the high degree of aging in China, polypharmacy may pose greater challenges to health outcomes and economic burden, due to the complexity of patients’ healthcare needs and frequent interactions with medical services that are fragmented, ineffective, and incomplete (Picco et al., 2016). Because of the insufficient implementation of the hierarchical medical system (Shen et al., 2018; Tang et al., 2021), it is common for elderly patients to see doctors in multi-institution and multi-department in China. However, prescribing is largely based on evidence-based guidance for a single disease, and often does not take into account the risk of polypharmacy (Molokhia and Majeed, 2017; Lin et al., 2020). Besides, according to China’s national survey, more than 90% of the elderly live at home. Among this elderly group the vast majority, with clear diagnoses of chronic diseases and somewhat stable conditions, chooses to take medications at home, and more than 27% of the elderly chooses the community or township medical institutions for treatment (China National Working Committee Office on Ageing, 2018). Therefore, it is particularly important to early identify the community-dwelling elderly at high risk of polypharmacy through community medical institutions, to carry out appropriate medication reconciliation.

Significant progress has been made on risk factors for polypharmacy. For example, age, living areas, number of diagnoses, number of visits, preference for medical institutions, etc., may increase the risk of polypharmacy (Halli-Tierney et al., 2019; Ishizaki et al., 2020; Balkhi et al., 2021). Based on the above factors, we can identify high-risk groups for polypharmacy and implement targeted disease prevention measures. However, there is no well-recognized risk prediction tool for polypharmacy of community-dwelling elderly patients.

The nomogram is a novel risk prediction model based on multivariate logistic analysis with multiple indicators. Several studies have shown that it is widely used for risk prediction of various diseases, carrying significance for screening and clinical practice (Han et al., 2018; Kim et al., 2021; Pan et al., 2021). In this study, a risk prediction model of polypharmacy represented by a nomogram for community-dwelling elderly patients based on the Chinese population was constructed.

## Materials and methods

### Study design and patients

A cross-sectional study was conducted in Shanghai by the Department of Health Policy and Management, School of Public Health, Fudan University. Based on simple random sampling in cross-sectional studies, the sample size to estimate the overall incidence rate was calculated as follows:

$$n=z\begin{array}{l} 2\\ \alpha /2 \end{array}{\pi \;\left(1-\pi \right)\;/\;\delta }^{2}$$

As the total population aged 65 and above in Shanghai was known, which was 3,616,600 as of 31 December 2019 (Shanghai Municipal Health Commission, 2019), the correction formula to calculate the sample size was used as follows:

$$n_{c}=n\;/\;\left(1+\frac{n}{N}\right)$$

The following formula could be obtained through integration:

$$n=\frac{N\cdot Z\begin{array}{l} 2\\ \alpha /2 \end{array}\;\pi \;\left(1-\pi \right)}{N{\cdot \delta }^{2}+Z\begin{array}{l} 2\\ \alpha /2 \end{array}\;\pi \;\left(1-\pi \right)}$$

Due to the large population, the allowable error is 0.1 and *a* is 0.05. According to the previous survey (Zhang et al., 2003), the daily incidence of polypharmacy among the elderly in a district of Shanghai (2,985 respondents) was 2.52%, so the calculated sample size was 92,000. The Shanghai Municipal Health Commission has the related data on known diagnoses of the entire Shanghai population from public medical institutions, while the Shanghai Medical Insurance Administration Center has related data on medication of the entire Shanghai population. We randomly extracted the diagnostic data of 92,000 patients aged 65 and above in 2019 from Shanghai Municipal Health Commission, and obtained the outpatient medication data of the above-mentioned patients from the Shanghai Medical Insurance Administration Center. After matching, there were 86,232 patients with valid diagnosis and medication data in 2019 (the effective rate was 93.73%).

In addition, cancer patients were excluded because they are beyond the specific scope of our study. Ultimately, except for the patients who visit the department of Oncology, 80,012 patients were included in the final sample.

Given the data source of the information system database of health government departments, the following ethical measures were implemented in data collection and analysis: 1) A confidentiality agreement was signed and does not allow the database to be shared in any form; 2) The database received by the research group is anonymous, therefore it excludes name, ID card, medical insurance card, home address, contact information, etc.; 3) All personnel in the research group who have access to the database are prohibited from online processing of the database, and need to use the office computer for data analysis without an external network. This study was approved by the Ethics Committee of School of Public Health, Fudan University (International Registration Number: IRB00002408 & FWA00002399; approval number: IRB#2021-11-0931).

### Variables and grouping criteria

The outcome variable, polypharmacy, was defined as the simultaneous use of five or more medications (excluding Chinese traditional medicine) for at least the last week.

For the independent variables, based on previous research results (Jokanovic et al., 2015; Yang et al., 2015; Tang et al., 2021; Tang et al., 2022), we used age, living district (countryside or suburb or central-city), preference for medical institutions, number of visits (for community healthcare center, secondary hospital, and tertiary hospital, respectively), number of diagnoses, main types of disease as the eight risk factors.

There are 3 types of living districts: countryside, suburb, and central-city. As for medical institutions, community healthcare centers, secondary hospitals, and tertiary hospitals were included, and the preferred institution was defined as the type of institution in which the patient had the greatest number of visits. Number of visits were grouped as follows: less than or equal to 5, ranging from 6 to 10, and greater than 10. The main type of disease was defined as the disease with the highest number of recurrences or, in case of a tie, the disease with the highest duration. According to the ICD-10 (10th revision of the International Classification of Diseases), including the circulatory system (I00-I99), the digestive system (K00-K93), the respiratory system (J00-J99), the musculoskeletal system and connective tissue (M00-M99), endocrine, nutritional and metabolic diseases (E00-E90), etc.

### Statistical analysis

R software (version 4.2.0) was used for statistical analysis.

Initially, 80,012 participants were randomly divided into a training set (60,010 participants) and a validation set (20,002 participants) at a ratio of 3:1 using the R “caret” package. Specifically, a series of test/training partitions are created using “createDataPartition” while “createResample” creates one or more bootstrap samples. The “createFolds” splits the data into k groups while “createTimeSlices” creates a cross-validation split for series data. The “groupKFold” splits the data based on a grouping factor. The mean ± standard deviation was used to describe data with a normal distribution, and number and percentages were used for categorical values.

Least Absolute Shrinkage Selection Operator (LASSO) regression is a contraction and variable selection method for regression models that shrinks the regression coefficient of certain variables to zero by imposing constraints on model parameters. Thus, the LASSO method was used to analyze data from the training set to select the best predictors of polypharmacy. The above included the eight variables used for the preliminary screening of risk factor variables. By introducing the selected features in the LASSO regression model, we constructed the prediction model by multivariate logistic regression analysis, and a *p*-value <0.05 was considered significant. The statistically significant predictors in the two groups were selected to establish the risk prediction model for polypharmacy, representing a nomogram.

In addition, the accuracy of the risk prediction model was estimated based on several validation methods by using the data of the training set and validation set. The receiver operating characteristic curve (ROC) was used to identify the quality of the nomogram to distinguish true positives from false positives based on the area under the curve (AUC). The calibration curve was drawn and calculated to evaluate the calibration of the polypharmacy risk nomogram. According to the net benefits of different threshold probabilities, the decision curve analysis (DCA) was used to determine the clinical utility of nomograms in this population.

## Results

### Characteristics of participants

A total of 80,012 patients with an average age of 75.29 ± 7.73 years were included in this study, of whom 50.06% were males. The patients’ mean number of diagnoses was 3.50 ± 1.99, receiving a median number of 6.10±4.02 drugs. The prevalence of polypharmacy (≥5 medications) was 60.13%, and the prevalence of extreme polypharmacy (≥10 medications) was 14.49%. Based on the ICD-10, the most frequent types of disease were in the circulatory system (35.27%), digestive system (10.04%), and respiratory system (7.22%). There were significant differences in age, sex, living district, preference for medical institutions, number of visits, number of diagnoses, and main types of disease between patients with polypharmacy and non-polypharmacy (*p* < 0.0001). After randomization, there was no statistical difference between the training set and the validation set. The homogeneity between the two sets supports the credibility of the model construction and related verification. Table 1 displays the characteristics of the study population according to different groups.

**TABLE 1:** Characteristics and factors of the participants.

Characteristics	Participants (*n* = 80,012)	Polypharmacy (*n* = 48,110)	Non-polypharmacy (*n* = 31,902)	*p*-value^*^	Training set (*n* = 60,010)	Validation set (*n* = 20,002)	*p*-value^#^
Age^†^	75.29 (7.73)	75.97 (7.95)	74.27 (7.26)	<0.0001	75.29 (7.72)	75.30 (7.75)	0.33
Sex				<0.0001			0.40
male	40,052 (50.06)	23,638 (49.13)	16,414 (51.45)		30,092 (50.14)	9,960 (49.80)	
female	39,960 (49.94)	24,472 (50.87)	15,488 (48.55)		29,918 (49.86)	10,042 (50.20)	
Living district				<0.0001			0.54
countryside	15,926 (19.91)	8,980 (18.67)	6,946 (21.77)		11,991 (19.98)	3,935 (19.67)	
suburb	35,465 (44.32)	21,505 (44.70)	13,960 (43.76)		26,542 (44.23)	8,923 (44.61)	
central-city	28,621 (35.77)	17,625 (36.63)	10,996 (34.47)		21,477 (35.79)	7,144 (35.72)	
Preference for medical institutions				<0.0001			0.71
community healthcare center	53,577 (66.96)	30,007 (62.37)	23,570 (73.88)		40,136 (66.88)	13,441 (67.20)	
secondary hospital	17,791 (22.24)	12,350 (25.67)	5,441 (17.06)		13,373 (22.29)	4,418 (22.09)	
tertiary hospitals	8,644 (10.80)	5,753 (11.96)	2,891 (9.06)		6,501 (10.83)	2,143 (10.71)	
Number of visits to the tertiary hospitals				<0.0001			0.33
≤5	65,050 (81.30)	36,067 (74.97)	28,983 (90.85)		48,854 (81.41)	16,196 (80.97)	
6–10	7,879 (9.85)	6,098 (12.67)	1781 (5.58)		5,890 (9.82)	1989 (9.95)	
>10	7,083 (8.85)	5,945 (12.36)	1138 (3.57)		5,266 (8.77)	1817 (9.08)	
Number of visits to the secondary hospital				<0.0001			0.98
≤5	51,252 (64.05)	25,199 (52.38)	26,053 (81.67)		38,432 (64.05)	12,820 (64.09)	
6–10	12,575 (15.72)	9,203 (19.13)	3,372 (10.57)		9,429 (15.71)	3,146 (15.73)	
>10	16,185 (20.23)	13,708 (28.49)	2,477 (7.76)		12,149 (20.24)	4,036 (20.18)	
Number of visits to the community healthcare center				<0.0001			0.29
≤5	24,951 (31.18)	11,491 (23.89)	13,460 (42.19)		18,764 (31.27)	6,187 (30.93)	
6–10	16,072 (20.09)	8,444 (17.55)	7,628 (23.91)		12,099 (20.16)	3,973 (19.86)	
>10	38,989 (48.73)	28,175 (58.56)	10,814 (33.90)		29,147 (48.57)	9,842 (49.21)	
Number of diagnoses^†^	3.50 (1.99)	3.78 (2.06)	3.07 (1.79)	<0.0001	3.50 (1.99)	3.50 (1.99)	0.25
Number of drugs^†^	6.10 (4.02)	8.22 (3.86)	2.90 (1.01)	<0.0001	6.09 (4.00)	6.12 (4.05)	0.37
Main types of disease				<0.0001			0.84
Circulatory system	28,221 (35.27)	19,618 (40.78)	8,603 (26.97)		21,144 (35.23)	7,077 (35.38)	
Digestive system	8,037 (10.04)	4,685 (9.74)	3,352 (10.51)		6,005 (10.02)	2032 (10.16)	
Respiratory system	5,774 (7.22)	4,046 (8.41)	1728 (5.42)		4,363 (7.27)	1411 (7.05)	
Musculoskeletal system and connective tissue	3,357 (4.20)	1831 (3.80)	1526 (4.78)		2,528 (4.21)	829 (4.14)	
Endocrine, nutritional and metabolic diseases	2,936 (3.67)	1925 (4.00)	1011 (3.17)		2,187 (3.64)	749 (3.74)	
Others	31,687 (39.60)	16,005 (33.27)	15,682 (49.15)		23,783 (39.63)	7,904 (39.52)	

^*^ *p*-value means the differences associated with polypharmacy.

^#^ *p*-value means the differences between the training set and the validation set.

^†^ The data in this marked part are presented as mean (SD), and the others as frequencies (%).

### Polypharmacy risk prediction model construction in the training set

**FIGURE 1:**
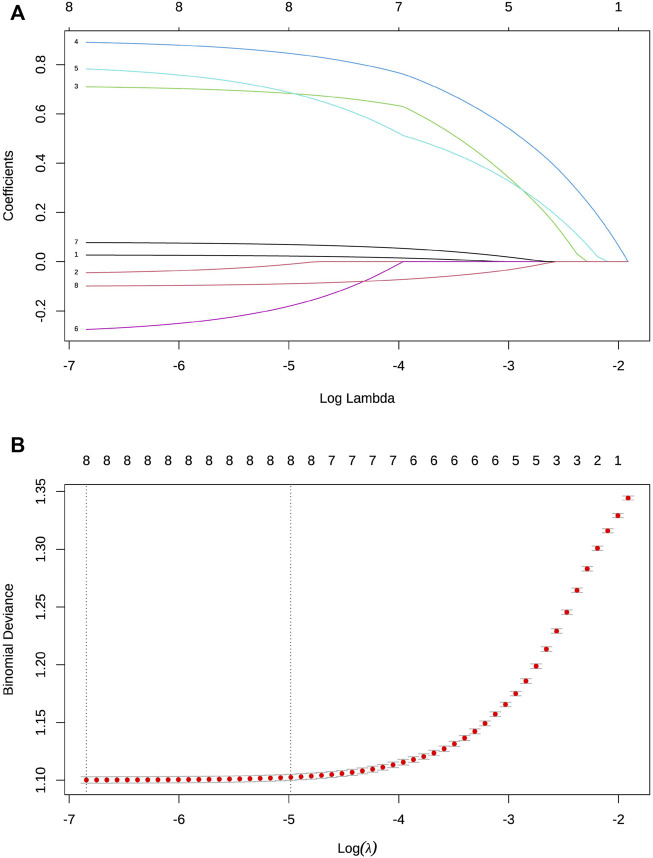
Selection of variables by LASSO binary logistic regression model. **(A)** Each curve in the figure represents the shifting track of each independent variable coefficient. The *y*-axis is the value of the coefficient, the top *x*-axis is log (λ), and the bottom *x*-axis is the number of non-zero coefficients in the model at this time. All the eight variables with non-zero coefficients were selected by deducing the best λ. **(B)** Based on cross-validation, each λ value can be used to obtain the confidence interval of a target parameter. Two dashed lines indicate two special λ values: one represents the mean value of the minimum target parameter, and the other represents the model with excellent performance but the minimum number of independent variables.

LASSO regression analysis was used to select the predictor variables, and multivariate logistic regression was used to establish the prediction model. All the eight variables, including age, residential area, preferred medical institutions, number of visits to tertiary hospitals, number of visits to secondary hospitals, number of visits to community health centers, number of diagnoses, and main types of disease, were included in the risk prediction model, since they had nonzero coefficients in the LASSO regression model (Figure 1).

**FIGURE 2:**
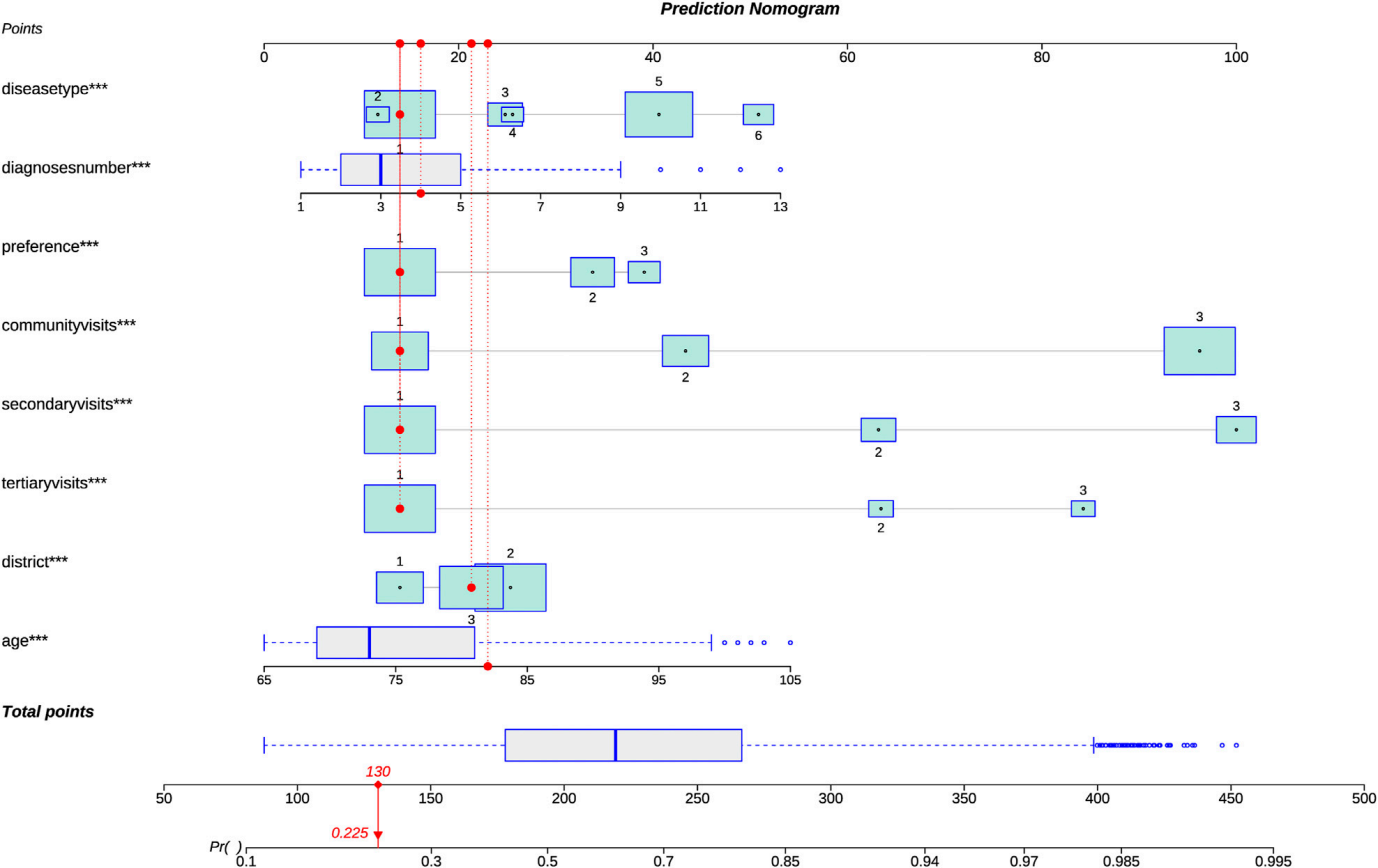
Risk prediction model for polypharmacy in the elderly (nomogram). Because there were significant statistical differences among these eight predictors, we introduced them into the prediction model to establish the polypharmacy risk nomogram, which is used to quantitatively predict the polypharmacy risk probability in the elderly population. For types of living districts, 1 represents countryside, 2 represents suburb, and 3 represents central-city. For the preference for medical institutions, 1 represents community healthcare centers, 2 represents secondary hospitals, and 3 represents tertiary hospitals. For the number of visits, 1 represents less than or equal to 5, 2 represents ranging from 6 to 10, and 3 represents greater than 10. For the main types of disease, 6 represents the respiratory system (J00-J99), 5 represents the circulatory system (I00-I99), 4 represents endocrine, nutritional and metabolic diseases (E00-E90), 3 represents the digestive system (K00-K93), 2 represents the musculoskeletal system and connective tissue (M00-M99) and 1 represents others. For example, by using the nomogram model, it could be concluded that a 82-year-old man, living in the central-city area, diagnosed with four diseases, and the main type of diseases is none of J00-J99, I00-I99, E00-E90, K00-K93 and M00-M99, with the numbers of visits less than or equal to 5 at different levels of medical institutions respectively, with the preference for community healthcare centers, had a 22.5% risk of developing polypharmacy.

The results of the multiple logistic regression analysis of these eight variables are shown in Table 2. Higher age, more frequent visits, more kinds of diseases, and having common diseases were risk factors for polypharmacy while living in the countryside and preference for community healthcare centers were the protective factors. Because these eight predictors were statistically significant variables, we introduced them into the prediction model to establish the polypharmacy risk nomogram, which is used to quantitatively predict the polypharmacy risk probability in the elderly population (Figure 2).

**TABLE 2:** Logistic regression analysis of risk predictors for polypharmacy in the elderly based on training set.

Variables	Coefficient	Multivariate *p*-value	OR (95%CI)
Age	0.027	<0.0001	1.03 (1.02–1.03)
Living district		<0.0001	
Countryside	1		1
Suburb	0.214		1.24 (1.18–1.30)
Central-city	0.130		1.14 (1.08–1.20)
Preference for medical institutions		<0.0001	
Community healthcare centers	1		1
Secondary hospitals	0.467		1.60 (1.48–1.72)
Tertiary hospitals	0.545		1.72 (1.59–1.88)
Number of visits to the tertiary hospitals	−0.745	<0.0001	0.48 (0.45–0.50)
Number of visits to the secondary hospital	−0.838	<0.0001	0.43 (0.42–0.45)
Number of visits to the community healthcare center	−0.844	<0.0001	0.43 (0.42–0.44)
Number of diagnoses	0.082	<0.0001	1.09 (1.07–1.10)
Main types of disease		<0.0001	
Respiratory system	0.719		2.05 (1.89–2.23)
Circulatory system	0.518		1.68 (1.60–1.76)
Endocrine, nutritional and metabolic diseases	0.283		1.33 (1.20–1.47)
Digestive system	0.213		1.24 (1.16–1.33)
Musculoskeletal system and connective tissue	−0.017		0.98 (0.89–1.08)
Others	1		1

### Polypharmacy risk prediction model verification in both sets

**FIGURE 3:**
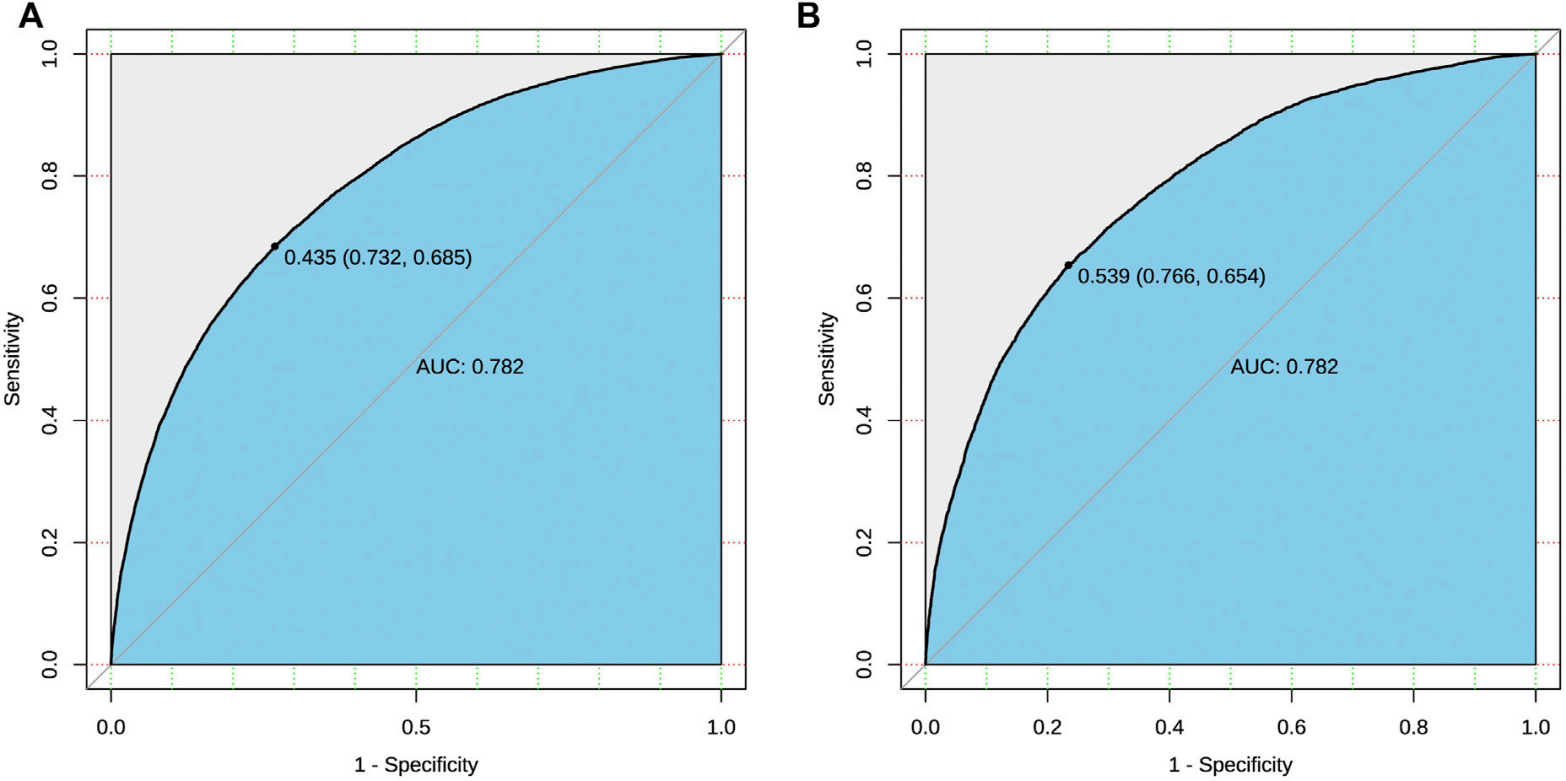
ROC curve of the risk prediction model for polypharmacy in the elderly. The black bold line represents the performance of the nomogram in both sets respectively [**(A)**: training set; **(B)** validation set]. The *y*-axis represents the true positive rate of risk prediction, and the *x*-axis represents the false positive rate of risk prediction.

ROC curve was used to evaluate the discriminant ability of the prediction model. For the prediction model, the AUC of the nomogram was 0.782 in both sets, demonstrating that the model has good performance (Figure 3). AUC greater than 0.75 indicates that the model has enough discrimination to correctly judge the results of dependent variables.

**FIGURE 4:**
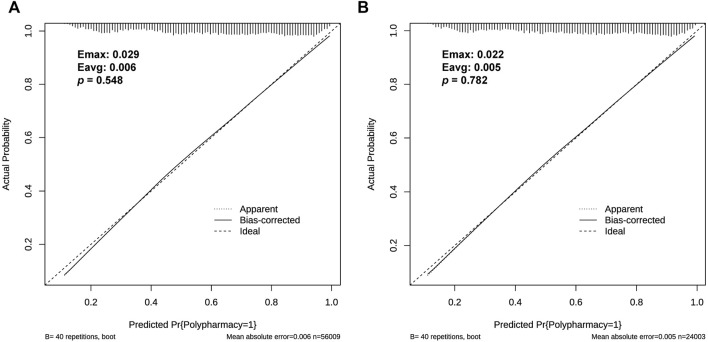
Calibration curve of the risk prediction model for polypharmacy in the elderly. The *y*-axis represents the actual diagnosed cases of polypharmacy, and the *x*-axis represents the predicted risk of polypharmacy. The black solid lines above the *x*-axis represent the sample distribution. The dotted line on the diagonal indicates the perfect prediction of the ideal model, and the solid line indicates the performance of both sets respectively [**(A)**: training set; **(B)** validation set]. *P* > 0.05 means that the calibration test has passed. The closer the solid line is to the dotted line, the better the predictive effect is.

Calibration charts were used to check the calibration of the prediction model, showing that the prediction model fits well with the validation set. The Hosmer-Lemeshow test shows that the predicted probability was highly consistent with the actual probability (training set, *p* = 0.548; validation set, *p* = 0.782) (Figure 4). A *p*-value greater than 0.05 means the model could estimate and fit the data at an acceptable level, and if greater than 0.1 indicates the model has a high prediction ability.

**FIGURE 5:**
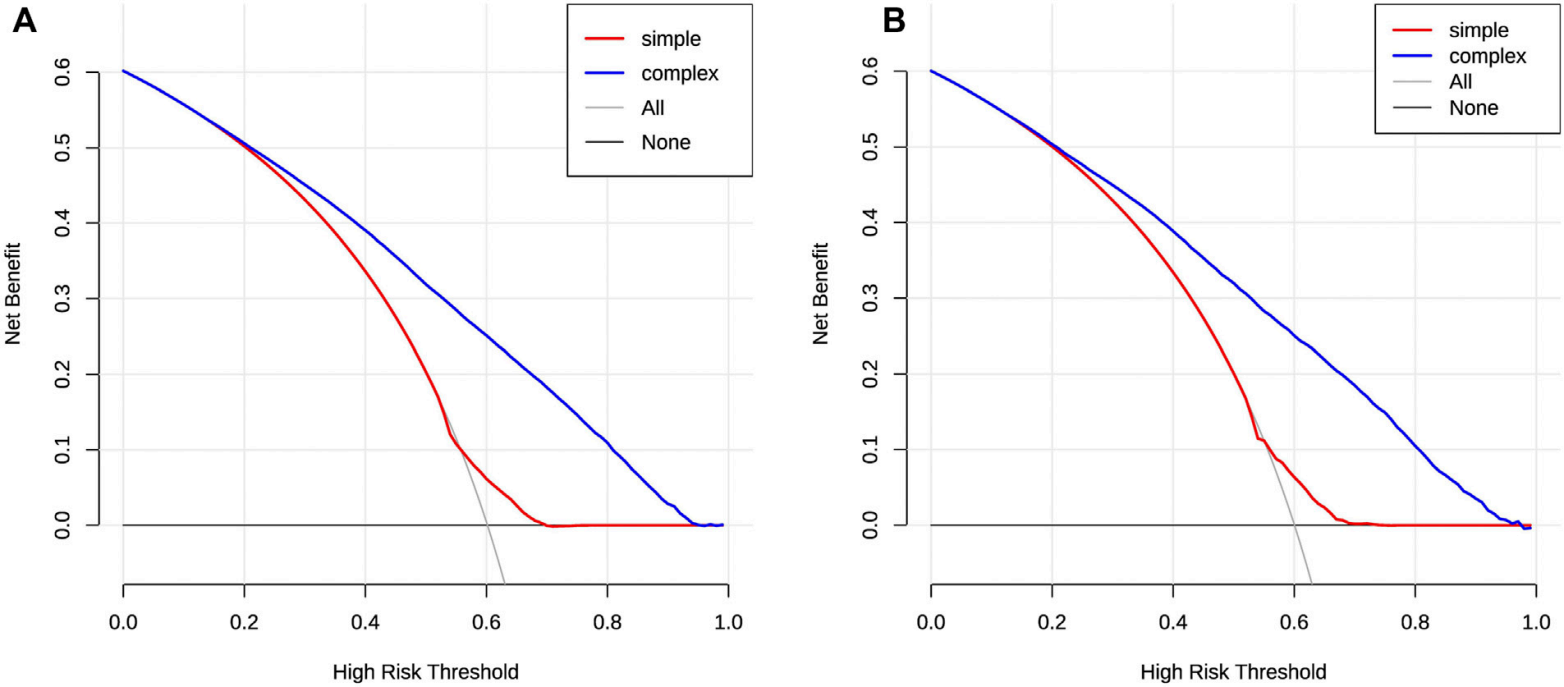
DCA of the risk prediction model for polypharmacy in the elderly. The *y*-axis is the net benefit, and the *x*-axis is the threshold probability. The black solid line indicates the assumption that non-participants have polypharmacy, and the gray solid line indicates the assumption that all participants have polypharmacy. The blue thick solid line represents the composited model, combined with the eight factors. The red thick solid line represents a simple model that contains only a single risk factor. In general, the farther the model curve is from the xy-axis, the stronger its clinical practicability is [**(A)**: training set; **(B)** validation set].

DCA results display that the threshold probabilities of the two sets in the prediction model reach up to 90%, implying that the model had a good application value (Figure 5).

## Discussion

Recognizing the scale of avoidable harm associated with unsafe medication practices, WHO launched the third Global Patient Safety Challenge: Medication Without Harm in March 2017 (Donaldson et al., 2017; Subakumar et al., 2021). Given that medicines are the most common treatment intervention, securing appropriate medication use should be considered a priority for countries committed to achieving the United Nations Sustainable Development Goals (SDGs).

As an economically developed area with a high degree of aging in China, the polypharmacy prevalence of Shanghai elderly patients included in this research was 60.13%. This study used institutional data from a representative sample of the elderly population in Shanghai, representing the polypharmacy level of the elderly in more developed regions of China. Other studies related to the prevalence of polypharmacy have shown different results. In a survey of 2,707 elderly European patients with an average age of 82.2 years, 51% used more than 6 drugs (Fialová et al., 2005; Onder et al., 2012); according to a survey of 300 thousand Koreans aged 65 and above, 86.4% of them have multiple drug use problems (Kim et al., 2014); a prospective surveillance study from India shown that polypharmacy prevalence was 45.0% among inpatients (Harugeri et al., 2010). Although there are some differences with other studies on polypharmacy prevalence, this difference can be interpreted considering methodological disparities in each study, such as polypharmacy definitions, patient ages, populations, data sources, etc. It is worth noting that, due to the complexity of Chinese traditional medicines, this study excluded them for analysis, so the actual situation of polypharmacy in the elderly may be more severe in reality.

Elderly patients have a variety of characteristics that make them more prone to polypharmacy. These indicators can help develop models for early identification of the patients at risk of polypharmacy and help to prevent polypharmacy along with its related problems. In this study, we built and validated a risk prediction model for polypharmacy with eight variables to identify elderly patients potentially at risk.

Greater age is an essential indicator [(Roe et al., 2002), (Park et al., 2016), (Pappa et al., 2011), (Hajjar et al., 2007)]. The nomograph formed in this study did not group the ages, but allowed the polypharmacy risk of the elderly to be more accurately evaluated according to their age.

The current study shows that elderly patients living in central areas are more likely to suffer from polypharmacy, which is related to China’s relatively loose referral system. With more medical institutions having better medical conditions and doctors in the central-city area, the elderly living in the central city could go to any specialized or general hospital for treatment in a closer geographical location, which increases the risk of polypharmacy, while for elderly patients living in the countryside more visits mean further transportation and higher costs.

Patients who prefer to see a doctor in the community have a lower risk of polypharmacy, which implies that medication review through primary pharmaceutical care based on the Family Physician Service optimizes the use of medicines for individual patients (Royal et al., 2006).

In addition, for any level of the medical institution, a lower number of patient visits is a protective factor against polypharmacy. In this study, the number of visits is grouped in the nomogram, which may weaken the accuracy of the prediction model, but it will broaden the practicality and applicability.

Undoubtedly, the number of diagnoses was a strong predictor of polypharmacy (Jörgensen et al., 2001; Junius-Walker et al., 2007). Research reveals that the risk of polypharmacy increased by 1.3 times for additional chronic diseases increase among the elderly in China (Yin and Zhang, 2012). At the same time, different disease types also have an independent impact on polypharmacy.

Given the convenience of application and high diagnostic performance, this study applied the nomogram to polypharmacy risk prediction. Based on the results of the above eight risk factors, we visualized the multivariate prediction model as a nomogram and verified it in three aspects of discrimination, calibration, and Clinical usefulness.

It should be considered that regular and comprehensive medication reviews, coordinated by the physician or pharmacist in primary care, are necessary interventions to keep polypharmacy under control (Nicieza-Garcia et al., 2016). The nomogram constructed in this study, which shows high clinical utility by DCA, can profitably be applied to community-dwelling elderly patients. Family physicians or pharmacists in primary medical units could scientifically and effectively use the model to screen the high-risk elderly with polypharmacy and carry out necessary prevention or intervention for those who may be at risk. This model is of great significance in the primary and secondary prevention of polypharmacy risk.

Several limitations of this study should be acknowledged. First, the data on the medication used in the study was institutional. Since the medical system is not completely interconnected, the drugs of private medical institutions are not involved, which means that the risk of polypharmacy is underestimated. Second, for the institutional data from sampling the elderly population, this study only collects the most basic demographic and medical history characteristics, lacking the sociological and economic characteristics of elderly patients. Nevertheless, the applied indicators are reliable and easy to obtain, so the model has high representativeness and strong applicability. Third, the model needs certain application conditions, such as economically developed areas, mature family physician services, pharmacist equipment, etc.

## Conclusion

In conclusion, this study explored the risk factors for polypharmacy among the elderly in Shanghai, China, and applied the nomogram to establish a predictive model *via* eight variables, which provided an effective tool for early screening and timely prevention of polypharmacy. Early identification of these patients, even before polypharmacy arises, is the first step in avoiding negative effects on their health. Family physicians or pharmacists could scientifically use the tool to closely observe community-dwelling elderly patients, decreasing the adverse health effects caused by improper medication use for the elderly in the future.

## Data Availability

The datasets presented in this article are not readily available because it has certain confidentiality. The analyzed contents generated during and/or analyses during the current study are available from the authors upon reasonable request. Requests to access the datasets should be directed to tangqi@fudan.edu.cn.
